# A critical exploration of nurses' perceptions of access to oncology care among Indigenous peoples: Results of a national survey

**DOI:** 10.1111/nin.12446

**Published:** 2021-08-02

**Authors:** Tara C. Horrill, Donna E. Martin, Josée G. Lavoie, Annette S. H. Schultz

**Affiliations:** ^1^ Nursing & Allied Health Research and Knowledge Translation BC Cancer Vancouver BC Canada; ^2^ College of Nursing, Rady Faculty of Health Sciences University of Manitoba Winnipeg Canada; ^3^ Department of Community Health Sciences, Rady Faculty of Health Sciences University of Manitoba Winnipeg Canada

**Keywords:** cancer, determinants of health, health inequity, health services accessibility, healthcare access, Indigenous health, Indigenous peoples, nurse roles, nursing, qualitative research, racism, social determinants of health

## Abstract

Inequities in access to oncology care among Indigenous peoples in Canada are well documented. Access to oncology care is mediated by a range of factors; however, emerging evidence suggests that healthcare providers, including nurses, play a significant role in shaping healthcare access. The purpose of this study was to critically examine access to oncology care among Indigenous peoples in Canada from the perspective of oncology nurses. Guided by postcolonial theoretical perspectives, interpretive descriptive and critical discourse analysis methodologies informed study design and data analysis. Oncology nurses were recruited from across Canada to complete an online survey (*n* = 78). Nurses identified a range of barriers experienced by Indigenous peoples when accessing oncology care, yet located these barriers primarily at the individual and systems levels. Nurses perceived themselves as mediators of access to oncology care; however, their efforts to facilitate access to care were constrained by the dominance of biomedicine within healthcare. Nurses' constructions of access to oncology care highlight the embedded narrative of individualism within nursing practice and the relative invisibility of racism as a determinant of equitable access to care among Indigenous peoples. This suggests a need for oncology nurses to better understand and incorporate structural determinants of health perspectives.

## 1 INTRODUCTION

Among Indigenous peoples in Canada,^1^ inequitable access to healthcare broadly, and oncology care specifically, have been well documented (Browne et al., 2011; Horrill et al., 2019; Horrill et al., 2020a; National Collaborating Centre for Aboriginal Health [NCCAH], 2011). Although access to oncology care is mediated by a range of factors (service location, provider availability, policies, social determinants of health), nascent evidence suggests that healthcare providers, including nurses, play a significant role in shaping equitable access to healthcare through their interactions with patients (Browne et al., 2016; Kitching et al., 2020; Wylie et al., 2019). From the perspective of Indigenous patients, experiences of racism, discrimination, or judgment on the part of healthcare providers have significant impacts on access to care (Browne et al., 2011; Cameron et al., 2014; Goodman et al., 2017). However, within the context of oncology care, the perspectives of these healthcare providers—particularly nurses, who are often the first point of contact for patients and provide the bulk of clinical care—have not been explored. In this paper, we discuss the findings of a study that used a postcolonial theoretical perspective to explore oncology nurses' understandings of access to oncology care among Indigenous peoples, and their perceived role in addressing access issues.

### 1.1 Problematizing our understanding of access

Access to care is most commonly understood as a function of the physical (geographical) location of healthcare services (Horrill et al., 2018). Physical access depends on the spatial and temporal distance between healthcare services and those accessing them (Wilson & Rosenberg, 2004). In Canada, approximately 48% of Indigenous peoples live outside of metropolitan areas, and 44% of registered First Nations peoples live on reserves (Statistics Canada, 2017). The remote and/or isolated geographical location of many Indigenous communities (including First Nations reserves) as a result of colonial government policies significantly impacts the inaccessibility of healthcare services (NCCAH, 2011). Beyond geography, the organization and appropriateness of services delivered and whether those services meet actual patient needs must also be considered (Pauly et al., 2009; Wilson & Rosenberg, 2004). Delays in diagnosis or treatment of illness, and unmet health needs such as untreated or under‐treated pain, indicate difficulties in accessing high‐quality, responsive healthcare services (Kitching et al., 2020).

In addition to physical distance, healthcare access is also often depicted as a function of service availability, where increasing health services is assumed to improve access to care. This ‘technocratic’ model of access is highly problematic with little regard for social barriers to accessing healthcare (Tang et al., 2015). A postcolonial perspective highlights several other important considerations beyond physical access (service location and availability), including structural and relational aspects of access. In particular, accessing healthcare requires that patients mobilize social, economic, and other resources in pursuit of healthcare services (Pauly et al., 2009; Wilson & Rosenberg, 2004). Increasingly, evidence points to the need to understand healthcare as a socio‐relational space, where access to care is constructed in part through the *interactions* between healthcare providers and patients (Horrill et al., 2018). Among Indigenous peoples, these interactions must be seen within the context of broader social and historical circumstances in which Indigenous peoples face the ongoing effects of colonialism through everyday experiences of racism and discrimination (Browne & Fiske, 2001). Indeed, experiences of racism in healthcare settings are one of the most significant barriers to accessing healthcare among Indigenous peoples (Allan & Smylie, 2015; Wylie et al., 2019).

### 1.2 Nurses and access to healthcare

As a framework for improving access to care among Indigenous peoples, cultural safety draws attention to unequal relations of power between patients and providers and challenges nurses to think critically about what they are bringing into each patient encounter (Anderson et al., 2003). A cultural safety perspective suggests that the attitudes of nurses, often the first healthcare professionals encountered, play a significant role in a patient's willingness to access healthcare (Papps & Ramsden, 1996). Nurses, therefore, can and do impact healthcare access. Within the Canadian context, healthcare encounters between nurses and Indigenous patients are often shaped by racism and discrimination, resulting in diminished quality of care and patients' reluctance to access healthcare services (Browne et al., 2011; Cameron et al., 2014; Goodman et al., 2017). Nurses' assumptions about Indigenous patients impact how patients' needs are understood, and how (or whether) those needs are met (Browne, 2007; Tang & Browne, 2008). This reflects the tendency among nurses practicing within Western contexts to focus on the individual patient while overlooking the socioeconomic, historical, or political context of patients' health and access to healthcare (Browne, 2005; Tang & Browne, 2008). However, research suggests that nurses are not necessarily *aware* of how their actions and interactions serve to produce/reproduce inequitable access to care (Browne, 2005; Tang et al., 2015). Despite this evidence, as a discipline, nursing remains largely silent on issues of racism and structural inequities (Thorne, 2017; Thurman et al., 2019).

### 1.3 Access to oncology care: Physical, structural and relational considerations

Oncology care can be defined as care related to the detection, treatment, or follow‐up (including rehabilitation, supportive, and palliative care) of cancer, regardless of setting, and is provided by a range of healthcare providers, including primary care providers, specialists, and allied healthcare providers. In Canada, responsibility for cancer control, including the planning and oversight of cancer services is given to provincial cancer agencies while the delivery of cancer services occurs through provincial, regional, and community cancer centers, tertiary care centers, and primary care settings.

Accessing oncology care presents many challenges for Indigenous peoples in Canada. Access to primary care, the entry point for gaining access to oncology care, is limited (Black, 2009; Olson et al., 2014; The Saint Elizabeth First Nations Inuit and Metis Program, 2012). Due to geographical location, cancer‐specific services, including cancer screening, are rarely available in Indigenous communities (Hammond et al., 2017; Lavoie et al., 2016; The Saint Elizabeth First Nations Inuit and Metis Program, 2012). As a result, patients must often travel long distances to access oncology care, relying on transportation that may not be feasible nor affordable (CancerCare Manitoba [CCMB], 2013; Galloway et al., 2020). These challenges are compounded by structural inequities, including poverty, access to housing, and food security (Assembly of First Nations, 2009; Black, 2009; Lavoie et al., 2016; The Saint Elizabeth First Nations Inuit and Metis Program, 2012). Negative healthcare experiences have also been documented from the perspective of Indigenous patients accessing oncology care, including experiences of racism, discrimination, and marginalization (Bottorff et al., 2001; CCMB, 2013; Macdonald et al., 2015). A history of oppression and assimilationist policies within Canada contribute to a documented distrust toward healthcare providers and institutions, further marginalizing Indigenous patients from the system purportedly meant to serve them (Bottorff et al., 2001; Maar et al., 2013).

Although research is limited, there is acknowledgment among healthcare providers that experiences of racism constitute a barrier to accessing oncology care, and conversely, that building relationships with patients is an essential aspect of delivering cancer services (Black, 2009; Maar et al., 2013; Wakewich et al., 2016). Although the relational aspects of care are key components of oncology nursing practice, these require adequate clinical time and support from leadership (Bakker et al., 2006; Davis et al., 2017). Within the context of oncology nursing practice, nurses are experiencing increasing patient acuity and workload, increasing complexity of the healthcare system, rapidly changing landscapes of cancer treatments, and multiple competing demands, all of which impact their ability to provide high quality, holistic care (Bakker et al., 2006; Davis et al., 2017; Raingruber & Wolf, 2015). However, to date, the perspectives of oncology nurses, who provide the bulk of clinical care to Indigenous patients with cancer, have not been heard. It is unclear whether these nurses understand the importance of relational aspects of care in the care of Indigenous peoples, or the impact of structural barriers on healthcare access. Moreover, while experiences of racism and their impact on access to oncology care are documented from the perspective of Indigenous patients, oncology nurses' perspectives on the impact of racism have not been studied.

## 2 METHODOLOGY

This paper presents the findings from one component of a doctoral thesis (TH) that critically examined access to oncology care among Indigenous peoples in Canada through the perspectives and professional roles of nurses. The multiple‐methods dissertation study included a national online survey conducted over a 6‐month period in 2019, the results of which are presented here. The specific research objectives for the online survey were to examine: (a) oncology nurses' perspectives of the barriers to accessing oncology care among Indigenous peoples, and (b) how oncology nurses understand their role in improving access. Informed by postcolonial theoretical perspectives, survey development and analysis of responses drew primarily upon interpretive descriptive (Thorne, 2016) and critical discourse analysis methods (Cheek, 2000). Ethical approval for this study was obtained from the University of Manitoba Education & Nursing Research Ethics Board, and from CancerCare Manitoba.

### 2.1 Theoretical perspectives

This study drew on postcolonial theoretical perspectives, which are rooted in analyses of power and colonial relations (Anderson et al., 2009). Research informed by postcolonial theories aims to produce transformative knowledge through the examination of the intersectional relationships between ‘race’, poverty, gender, and other factors, and by explicating the impact of these factors on health and healthcare delivery (Anderson, 2002; Browne et al., 2005). Importantly, the ‘post’ in postcolonial does not imply that colonialism is a thing of the past, but rather, denotes an imperative to work against and to move beyond colonialism, and to explicate the ways in which colonialism continues to reach both backward and forward (Anderson, 2004; McGibbon et al., 2014). Postcolonial scholarship aimed at exposing the extent to which colonial relations continue to shape the health and healthcare experiences of Indigenous peoples in Canada is essential to inform processes of decolonization in healthcare (McGibbon et al., 2014). As applications of postcolonial concepts in nursing practice, cultural safety and trauma‐ and violence‐informed care (TVIC) were also incorporated as sensitizing frameworks that drew attention to the impact of relationships between healthcare providers and patients on access to healthcare.

### 2.2 Sampling and recruitment

Canadian oncology nurses from diverse practice settings were recruited to participate in an online survey using convenience sampling strategies. Inclusion criteria included licensure as an active practicing registered nurse and a minimum of one year of current or previous oncology nursing experience. Nurses were recruited primarily by email through third‐party organizations, which contained an anonymous link to the online survey. Nurses who completed the survey could enter to win one of five $100 gift cards. An estimated 1450 invitations to participate were extended; unfortunately, we had no ability to determine how many were received and/or read.

### 2.3 Data collection

Using Qualtrics® online survey software, demographic information and responses to open‐ended questions were collected anonymously; informed consent was obtained electronically. Participants were required to read through an Informed Consent Form describing the study and their involvement. Participants were instructed to click “I agree” and “Continue” if consent to participate was granted, and to print or take a screenshot of the consent for their records. Participants were considered to have given informed consent if they proceeded through the consent form to complete and submit their survey responses. Following several demographic questions, a series of open‐ended questions, consistent with a qualitative research approach (Braun et al., 2020), invited respondents to share their experiences and perspectives on barriers to accessing oncology care among Indigenous peoples and ways in which they, as nurses, have or could facilitate access to oncology care for this population. As part of the survey, respondents watched a digital story of one Indigenous man's experiences of accessing oncology care, which was included to stimulate thinking about additional barriers to accessing oncology care at the systems and structural levels. Survey data was exported from Qualtrics® to Excel® (demographic data) and NVIVO® (qualitative data) for analysis.

### 2.4 Data analysis

Qualitative data from open‐ended responses were analyzed inductively and iteratively, consistent with an interpretive descriptive approach (Thorne, 2016). Survey responses were read repeatedly to gain a sense of the whole. Keywords, phrases, or ideas were identified in the data and used to form codes; patterns were then developed by clustering coded data into meaningful categories, which were collapsed and expanded as data analysis progressed (Thorne, 2016). NVivo® software was used during this stage to organize and sort raw and coded data. In the later stages, we moved toward a more abstract and conceptual analysis of the emergent themes to move beyond reporting descriptive findings and articulate clinical implications and applications of the research findings. Through the conceptual analysis, postcolonial theory drew attention to broader systems of power, underlying discourses, and other structural influences on access to care. Finally, cultural safety and TVIC frameworks guided the analysis to focus on relationships within healthcare and ways that nurses influenced access to care. The credibility of the findings was enhanced by the representativeness of the participants to oncology nurses in Canada (see below), and by the diversity of participants in terms of practice setting, professional roles, and levels of education and nursing experience. The findings were also critically appraised by two Indigenous healthcare providers with knowledge and experience of the oncology context. Throughout the analysis, an audit trail was maintained, with analytic and interpretive decisions documented (Thorne, 2016).

## 3 RESULTS

### 3.1 Survey response rate

A total of 81 responses were received from the invitation to participate in an online survey, for a response rate of approximately 6% of all invitations extended (not necessarily received, the number of which was not available). Of the 81 responses, three participants consented but did not answer any survey questions. An additional 78 participants answered some or all of the survey questions; a review of these partial responses suggests that as questions required more thought and reflection, participants abandoned the survey. Finally, 34 participants completed all survey questions. Both full and partial responses were included in our analysis.

### 3.2 Participants

Survey participants were primarily registered nurses with significant nursing experience (Table 1). Most nurses worked in provincial cancer centers and practiced in direct patient care roles. When asked how frequently they worked with Indigenous patients, the majority indicated either at least daily (*n* = 15) or weekly (*n* = 35). Nurses were asked to identify their ancestry: a small proportion self‐identified as Indigenous (First Nations, Métis, or Inuit; *n* = 5); however, most identified as nonindigenous.

**Table 1:** Characteristics of survey respondents

	All participants (*n* = 78)	Partial completions (*n* = 44)	Full completions (*n* = 34)
Nursing experience			
Average # years nursing	18	19	17
Average # years oncology nursing	11	11	11

	**# of responses (%)**	**# of responses (%)**	**# of responses (%)**
Type of nursing registration			
RN	73 (94%)	42 (95.5%)	31 (91%)
APN (Clinical Nurse Specialist, NP)	4 (5%)	2 (4.5%)	2 (6%)
Not specified	1 (1%)	0	1 (3%)
Highest level of professional education			
Diploma	19 (24.5%)	13 (30%)	6 (18%)
Undergraduate degree (i.e., BScN, BN)	50 (64%)	27 (61%)	23 (67.5%)
Graduate Degree (i.e., MN, MSc, PhD)	9 (11.5%)	4 (9%)	5 (14.5%)
Type of oncology practice setting^a^			
Provincial cancer center	60 (71.5%)	34 (74%)	26 (66.5%)
Satellite cancer center	6 (7%)	4 (9%)	3 (7.5%)
In‐patient setting	13 (15.5%)	6 (13%)	7 (18.5%)
Other	5 (6%)	2 (4%)	3 (7.5%)
Type of nursing position			
Direct patient care	58 (74%)	32 (73%)	26 (76.5%)
Non‐direct patient care (clinical resource, charge, manager)	20 (26%)	12 (27%)	8 (23.5%)
Frequency of working with Indigenous patients			
Daily (at least once per day)	15 (19%)	8 (18%)	7 (21%)
Weekly (at least once per week)	35 (45%)	23 (52%)	12 (35%)
Monthly (at least once per month)	13 (17%)	7 (16%)	6 (18%)
Occasionally (at least once per year)	15 (19%)	6 (14%)	9 (26%)
Ancestry			
Self‐identify as Indigenous (First Nations, Métis, Inuit)	5 (6.5%)	3 (7%)	2 (6%)
Self‐identify as other than Indigenous	68 (87%)	36 (82%)	32 (94%)
Not specified	5 (6.5%)	5 (11%)	0

^a^ Participants could indicate more than one practice setting.

Our sample was largely representative of oncology nurses in Canada, based on the membership statistics of the Canadian Association of Nurses in Oncology [CANO]. With a membership base of approximately 1100, CANO represents a large portion of oncology nurses in Canada (CANO, 2019). More than two‐thirds of its members are educated at the undergraduate level and most practice in direct patient care roles.

### 3.3 Survey results

Analysis of survey responses began with categorizing the types of barriers to accessing oncology care identified by nurses. We were interested in how nurses discussed these barriers—as individual, systems, or structural level barriers—as these narratives shed light on how nurses understand access to care, and how they might address barriers. When considering their role, nurses primarily saw themselves as *mediators* of access to oncology care, which at times was constrained by a biomedical model of nursing practice.

#### 3.3.1 Nurses' narratives of barriers to accessing oncology care

Nurses discussed multiple barriers to accessing oncology care for Indigenous peoples, their narratives primarily focusing on the actions or domains of individual patients or healthcare providers. Although some nurses talked about healthcare system barriers, few connected the individual and systems‐level barriers to societal structures underlying these barriers that result in inequitable access to oncology care for Indigenous peoples.

##### Individual

The barriers most commonly discussed by nurses included transportation, communication, financial, and psychosocial challenges. Nurses tended to construct these as barriers to accessing oncology care at the individual level, for example, speaking about barriers as “sometimes noncompliance; sometimes comprehension on antinausea meds, central lines; sometimes no shows on appointments” (S24), without fully acknowledging contextual factors shaping or creating these barriers.

Nurses described transportation as a significant barrier to accessing oncology care (S21, S26, S40, S61). Given the geographical location of many Indigenous communities and the lack of services available locally, many Indigenous patients must travel to larger urban centers to access care. At times, nurses' observations regarding transportation barriers took a paternalistic tone. For example, one nurse stated, “sometimes…patients are not aware that some appointments are time sensitive or should not be delayed” (S78), while another nurse highlighted a patient's “inability to attend appointments reliably” (S35). These narratives locate the ‘problem’ of transportation with the individual and their lack of ‘awareness’ rather than contextual factors beyond the individual that may be at play.

Communication was identified as problematic (S45, S52). Nurses often rely on telephone access to monitor the side effects of treatment and assess patient concerns; however, within the context of financial challenges and unreliable telecommunication services in some Indigenous communities, patients may not have consistent access to a telephone (for example, S21, S51). Nurses also identified language as a barrier to effective communication (S13, S22, S46, S70), and described frustration at their ability to conduct patient teaching when patients were not able to communicate in English (S55). Patient education presents real challenges within the context of oncology care, where treatments are complex, side effects may be significant, care is primarily provided on an outpatient basis and time with patients is limited. Through a postcolonial lens, it is likely that telephone access and language intersect with cultural differences, unequal relations of power, and the historical context of Indigenous patients, adding additional layers of complexity to communication challenges, which nurses in this study did not discuss.

Poverty and financial ability to afford expenses not covered under health insurance were highlighted as barriers (S35, S72); mental health issues and homelessness also impacted some patients' ability to attend appointments and complete treatment.

The patient was dealing with homelessness, chemical addictions, and had recently moved from a remote community to the city…this patient was not always reliable to show up for appointments (S35).

Lack of psychosocial supports, particularly among patients who relocated to urban centers for cancer treatment, meant some patients had to navigate the cancer treatment experience alone, resulting in tangible consequences, such as missed appointments or medications (S41, S75).

A small proportion of nurses highlighted how interactions between patients and healthcare providers can constitute barriers to accessing oncology care. Nurses described how a lack of understanding of cultural values among healthcare providers was problematic at times (S13, S40). One nurse explained:

The healthcare team really struggled to honor this patient and her family's time schedule, as it was quite different than how they conceptualized their time schedule…Often, this would result in frustration for the patient, the family, and the healthcare team. The barrier that this patient and family faced was a lack of education for healthcare staff about navigating the complex care needs of Indigenous people (S40).

Nurses recalled negative healthcare experiences of Indigenous patients, such as those whose health concerns were repeatedly dismissed by healthcare providers who did not take them seriously or assumed that reported symptoms were psychological rather than physical (S23, S70). Some nurses recognized these experiences as a barrier to accessing care, particularly in response to the digital story highlighting one patient's oncology care experiences. In several cases, this was explicitly labeled as racism (e.g., S56, S80), whereas other nurses used the terms discrimination or stereotyping that ‘could be’ interpreted as racism (S03, S38). A few nurses indicated that the dismissal of patient concerns and experiences of racism impacted access to timely and high‐quality care. One nurse relayed a particularly upsetting experience.

The patient…had been mis‐diagnosed until his cancer presented as stage 4, and he was in constant pain. He had trouble numerous times with his pharmacy, as they didn't want to dispense his pain meds because they felt that the dose prescribed was inappropriate and that he must be drug‐seeking. He was tearful and said ‘I wish I was white. Then I wouldn't have to be in pain'. His suffering was directly related to incorrect assumptions and racism (S23).

While there was some understanding of discrimination and racism as impacting access to care, this was uncommon and more often than not absent in survey responses. Moreover, although through a critical perspective, racism is understood as rooted in historical, political, and social structures, nurses in this study tended to convey an understanding of racism that was limited to the interpersonal level.

##### Systems

Nurses highlighted the complex and siloed nature of the Canadian healthcare system and the challenges navigating this system as significant barriers to accessing oncology care. Nurses indicated that difficulty accessing primary care can be a problem for some Indigenous patients, resulting in limited cancer screening opportunities and delays in cancer diagnoses (e.g., S03, S51, S56). However, several nurses perceived that despite challenges in accessing primary care, once patients are in the oncology system, there are no barriers to accessing oncology care: “after arriving at our hospital there were no barriers to treatment as every patient is categorized and assigned to a protocol that best treats their disease” (S18; see also S03, S08). Limited local availability of allied health professionals (e.g., dieticians) and specific services (e.g., diagnostics) was also noted to impact the quality and timeliness of care (S14, S21, S35).

##### Structural

Structural barriers to accessing healthcare are those that are embedded in the historical, economic, social, and political fabric of society. A few nurses identified social factors, including financial challenges, unemployment, homelessness, and lack of housing as barriers to accessing oncology care; however, few other structural barriers were identified (S21, S35, S55). Notably, although these factors are rooted in structures, they were most often framed as individual challenges. Although nurses did not discuss policies as barriers to access, several nurses emphasized the role nurses can play in advocating for policy change at all levels of government.

#### 3.3.2 Nurses roles as mediators of access to care

Most nurses saw themselves as active participants in the process of helping patients gain access to oncology care, working on behalf of patients to bridge the gaps in care. As intermediaries in access to care, nurses functioned as coordinators, navigators, advocates, and educators. Survey responses indicating that nurses see themselves as able to influence access to care suggest that nurses have, on some level, an implicit understanding of their currency or power within the healthcare system to act on behalf of their patients.

Nurses functioned as mediators of access to care by coordinating care on behalf of patients to ensure seamless delivery of care (S09, S25, S30, S40). Some nurses indicated that they were the ‘glue’ holding the multi‐disciplinary team together, and an essential liaison between community and oncology settings (S18, S25, S40, S75). Care coordination at times required significant investments of time and effort.

I was able to help him apply for Seniors Benefits to help pay for his meds and supplements. I was able to get social work involved and arrange for his wife to be in contact with a speech‐language pathologist when they returned to their community. I had to arrange for his diet supplements to be shipped to his community via bus (S14).

Nurses also guided patients in navigating the oncology system by troubleshooting barriers and connecting them to resources (S03, S04, S35, S50, S72). To aid in navigating the system, some nurses emphasized the need to “increase [our] own awareness of patients' contexts and healthcare services available in their community” (S75; see also S30, S77). Although nursing roles specific to patient navigation (including nurse navigators and Indigenous navigators) exist within most oncology settings in Canada, and many nurses discussed referring patients to a navigator, some nurses saw that supporting the patient to navigate the healthcare system was also part of their role (S02, S14, S18, S21, S30). One nurse described their role as “doing as much ‘leg work’ as I can. Providing any names, numbers, services etc.” (S35).

Common among many responses was the perspective that nurses play a role in “advocating for families and patients when they may not speak up for themselves” (S25). As advocates, nurses occupy a middle position between patients and other healthcare providers, institutions, systems, and governments. Some nurses highlighted specific ways in which they could advocate for individual patients, such as timely cancer diagnosis (S03, S18, S23, S38), while other nurses discussed a broader advocacy role (S23, S80). One nurse explained:

Nurses are ideally suited to ensure people have access to oncology care. As a profession, we have not always been encouraged to use our voice, particularly when it comes to political issues. However…nurses are well positioned to influence policy and improve care (S56).

Similarly, nurses function as intermediaries through their roles as educators, ensuring patients and families understand their diagnosis, treatment, and potential side effects (S14, S39, S41). Although most nurses discussed the importance of education out of concern that patients understand when and where to access care if needed (S26, S40, S56, S67), some took a more paternalistic approach (S35, S70). For example, one nurse noted how patients “need to be informed in their own health care” (S12). These narratives construct a binary of ‘us/them’ and tend to be used as a strategy to place responsibility for accessing care solely on the patient.

Developing relationships with patients was an important way for nurses to improve access to oncology care for Indigenous patients (S13, S42, S52, S56). A relational approach to care recognizes that needs extend beyond the physical, and that relationships within healthcare settings are an important component of nursing care. Although some form of nurse–patient relationship is implicitly needed for nurses to function as mediators of access as described above, some nurses discussed this more explicitly.

I think that relationship is important in caring for people who have barriers to access. Nurses are well poised to develop relationships with patients…relationships can provide a safe place in which to share concerns or other issues (S75).

Relationships are particularly important given the context of colonizing relationships within Canada between Indigenous peoples and institutions, and the resulting distrust that exists among Indigenous peoples toward healthcare institutions. Several nurses expressed the need to increase education on Indigenous history in nursing curriculums, with the recognition that understanding this history is one essential component of providing culturally safe care (S13, S75). Endeavoring to be sensitive to the patient's history, avoiding judgment, and making the patient feel safe were identified as ways to improve access to care (S35, S45, S69). As a result of developing trusting relationships, nurses could better understand and meet the needs of patients.

However, this perspective was not expressed by all nurses: “at the individual level, I can…help our patients feel safe in the system—however, making their experience better does not necessarily improve their access” (S14). Indeed, while many nurses saw themselves as mediators of access to oncology care, some nurses did not talk about themselves as having a role in mediating access to care. These nurses tended to identify *other* healthcare professionals (such as nurse navigators) or services (Indigenous‐specific services) as responsible for addressing any gaps in care or barriers to accessing oncology care (S22, S29, S61, S73, S76). Notably, those who did not see themselves as playing a role in facilitating access to oncology care worked less often with Indigenous patients and had fewer total years of nursing experience, which suggests they may not have a good understanding of the barriers Indigenous patients face.

**Biomedical Constraints and Nursing Practice**. Although many nurses saw themselves as playing a role in improving access to oncology care, they also felt constrained in their abilities to practice in ways known to improve access to care. To illustrate a common response among survey participants, this nurse expressed frustration at the lack of time for nursing care beyond patients' physical needs:

There isn't enough time in a busy tertiary cancer centre (which is over capacity) to get into detail about all the important aspects of our patients. It also takes trust to develop relationships, and this also takes time (S003).

Nurses also acknowledged that their lack of understanding of services needed or available for Indigenous patients with cancer (S002, S022, S035, S041, S074, S080), barriers faced (S024), and Indigenous history and perspectives on health (S042, S067, S075) impacts their ability to improve access to oncology care. Several nurses perceived a lack of power to make positive changes within a hierarchical healthcare system (S023, S045, S075). These concerns highlight how nursing practice is subject to the powerful influences of the biomedical and business models, both structural barriers to healthcare access.

## 4 DISCUSSION

This study explored oncology nurses' perceptions of access to oncology care among Indigenous peoples. This lens also provided an opportunity to appreciate where nurses' attention is focused within their practice and to understand their experiences caring for Indigenous Peoples. Many barriers to accessing oncology care identified in this study are similar to those previously acknowledged, which include limited access to primary and oncology care locally, difficulties accessing or arranging transportation and accommodations, language and lack of interpretation services, inadequate financial resources, and lack of support for patients who relocate for cancer treatment (Black, 2009; CCMB, 2013; Hammond et al., 2017; Howard et al., 2014; Lavoie et al., 2016; Macdonald et al., 2015; Olson et al., 2014; The Saint Elizabeth First Nations Inuit and Metis Program, 2012). Notable discourses operating within the nurses' responses included narratives of individualism, resulting in limited insight into structural contexts shaping access to care, and the absence of acknowledging racism as a barrier to accessing care. While some nurses spoke about the importance of relationships and trust‐building, the key roles oncology nurses saw themselves playing to improve access to care were through care coordination, education, advocacy, and relationship‐based care with individual patients. However, nurses also talked about the hegemonic biomedical model, which constrained their ability to practice in ways that maximize access to care and provide high‐quality care.

The diverse barriers identified by nurses are reflections of those well documented to be shaping Indigenous peoples experiences with accessing oncology care. This study advances the knowledge about how these barriers are understood among oncology nurses and therefore, most likely to be addressed. Findings suggest that oncology nurses construct barriers to accessing care primarily as barriers at the individual and systems levels. In some ways, this is not surprising and reflects a strong commitment to individualism within Western societies, along with the dominance of biomedicine in healthcare and on nursing as a discipline. Individualism understands humans as abstract from their context, with successes and failures (health and illness, for example) attributed to individual character rather than social structures (DiAngelo, 2018). The biomedical model and its focus on the individual are woven into the fabric of nursing within Western contexts (Browne, 2001; Hilario et al., 2018), and within this frame, nurses tend to know and speak about access to care as an individual responsibility. In the current study, this was reflected through nurses' statements that “noncompliance”, “no shows for appointments,” and ability to afford medications were barriers to accessing oncology care. While these may be important issues to address, there are significant structural inequities shaping a patient's ability to ‘comply’ with treatment, ‘show up’ for appointments, or ‘afford medications’ that were *not* spoken about in the survey responses. However, a well‐meaning, nursing practice focused on the individual will fail to adequately address broader issues of marginalization, social inequities, and structural determinants of health and risks further entrenching patterns of inequitable access to care (Browne, 2001; Raphael et al., 2008).

The influence of biomedicine was also evident in nurses' complaints regarding a lack of time to develop trusting relationships, provide teaching and supportive care, and address the broader context of patients' health. These concerns point to a system that prioritizes time‐ and cost‐efficiency over high‐quality nursing care. The stated limited understanding among nurses about Indigenous ways of knowing, histories of colonization, and healthcare needs ought to be interpreted within the context of a powerful biomedical model that pushes nursing education and practice toward assessing and addressing physiological needs abstract from broader social and historical contexts. The fragmented nature of the current healthcare system also highlights the biomedical propensity to treat bodily parts and diseases as disintegrated and separated from the whole person. Thus, the biomedical and business models, and the ways in which healthcare is structured as a result, become significant barriers to accessing care. Although these systemic and structural barriers were not always visible to nurses, their influences were felt.

The demonstrated lack of awareness regarding structural influences on access to oncology care is concerning, and yet consistent with results from a recently published scoping review (Horrill et al., 2019). Many nurses in this study recognized their ability to practice in ways that improve access to oncology care for Indigenous peoples, yet these were primarily strategies to improve care for an individual patient rather than strategies to address systems or structures. Although the structural nature of some of the barriers to accessing oncology care was at times implied, it seemed as though nurses lacked the language needed to discuss these barriers as structural rather than the individual. Alternatively, nurses' social position, particularly among those who are white, allows them to remain oblivious to structures of inequity (Allen, 2006; Hall & Fields, 2013; McGibbon et al., 2014).

This is not entirely surprising given that although *social* determinants of health framework have been increasingly embraced over the last several decades, a focus on *structural* determinants of health and healthcare access has not been sustained in clinical practice or nursing education (McGibbon et al., 2014; van Herk et al., 2011). Until recently, concepts such as cultural safety, which facilitates reflection on social positioning and structural inequity, have not been included in nursing curricula or standards of practice in Canada (Baba, 2013). Understanding how health and healthcare are situated in social, political, and historical structures is critical for nurses to improve the quality of care and access to care (Beavis et al., 2015; McGibbon et al., 2014). Structural competency education has been proposed as one strategy to improve the ability of clinicians to see the clinical and patient impacts of ‘upstream’ or structural determinants of health (Metzl & Hansen, 2014). Incorporating education on structural determinants of health into nursing curricula and continuing education is imperative to ensure that nurses are adept in recognizing, understanding, and discussing structural influences on access to care, which could open space for nurses to consider how they could address these determinants of health.

One finding of particular interest was the notable absence of racism found within nurses' narratives of access to oncology care. Despite extensive evidence over the last several decades that Indigenous peoples experience racism and discrimination in healthcare encounters that seriously compromise access to care, nurses' perspectives on barriers to accessing care rarely included racism. Narratives tended to focus on barriers at the individual patient level, with very little acknowledgment of how systemic racism impacts access to care. Moreover, even when explicitly named and described within the digital story (as part of the survey), only 11 nurses identified racism as a barrier to accessing care. van Herk and colleagues (2011) argue that racism is not often considered an important issue within nursing, and issues of racism, power, and oppression have historically not been taught within professional nursing education. Thus, it is possible that oncology nurses lack understanding or awareness of how racism operates within healthcare settings and its impact on access to care. The absence of racism as a key finding does not necessarily represent the perspectives of *all* nurses who participated in this study, nor that nurses are *intentionally* avoiding discussions of racism – indeed some nurses did identify racism (both interpersonal and structural) as problematic within healthcare settings. However, the silence around racism within healthcare settings is not an unknown, and in an effort to understand why that might be, we offer three discourses that operate to sustain the invisibility of racism: political correctness, egalitarianism, and the good/bad binary (see Table 2).^2^

**Table 2:** Discourses sustaining the invisibility of racism

Discourse	Description	Example	Effect
Political correctness	Avoiding discussing or naming racism because it is politically incorrect (Henry & Tator, 2006; Hilario et al., 2018) or socially taboo (Tang & Browne, 2008)	Reluctance/hesitancy/discomfort in naming racism as a barrier; using hedging language: “Provider bias, *maybe* even prejudice/racism on behalf of the primary care physician” [emphasis added] (S003).	Ignores or downplays the prevalence of racism within healthcare settings
Egalitarianism	Purporting to treat all patients the same (Browne, 2005, 2007; Tang & Browne, 2008) Implying that if all patients are treated equally, they must be treated fairly (Henry & Tator, 2006) Presenting healthcare institutions as “discrimination‐free” (Tang & Browne, 2008)	“I treat patients with an Indigenous background daily…these particular patients are treated the same as every patient within [cancer center]. They are given the most up to date options for their cancer treatment and treated with care and compassion” (S039)	Denies existence of racism at the individual and structural levels within healthcare contexts – ‘we cannot be racist because we treat everyone the same’
Good/Bad binary (DiAngelo, 2018)	Understanding racism as specific ‘acts’ committed by ‘bad’ people, rather than as *systematically* and *structurally* produced; denying the possibility that ‘good’ people can participate in racism (DiAngelo, 2018) Racism within healthcare settings becomes an example of unprofessional behavior rather than an expression of ideologies embedded within social structures (Hilario et al., 2018)	Identifying behaviors or specific interactions between *other* healthcare providers and patients as racism (racism is a problem among healthcare providers in nursing stations or emergency rooms) Little acknowledgment of how these acts are situated within and produced by comprehensive systems of racism	Obscures the ways in which it operates through systems and structures to shape access to oncology care

Finally, although many nurses expressed an understanding of their roles as individual nurses and of the role of nursing as a profession in improving access to oncology care, notably, a small proportion of nurses did *not* see themselves as playing a role in access. This viewpoint may be influenced by an understanding of healthcare access as premised on geographical location and availability of services, and a view that nurses have little impact on these broader systemic factors. Alternatively, this view may suggest a poor understanding of the impact of trusting patient‐provider relationships on access to healthcare and oncology care. Incorporating principles of cultural safety and trauma‐ and violence‐informed care into nursing education and practice would highlight the importance of relational care (Horrill et al., 2020b). Cultural safety has been noted to be a key element of providing high‐quality, accessible healthcare for Indigenous Peoples (Browne et al., 2016; Horrill et al., 2020b). Integrating cultural safety into practice requires us as nurses to engage in reflexive processes and get uncomfortable with our own biases and attitudes, our shared history of colonialism in Canada, and how we may have been complicit in perpetuating structural inequities (Papps, 2015). Cultural safety, with its focus on unequal relations of power and patient‐provider interactions, will help us as nurses to develop an awareness of how racism is operating within healthcare settings, at both the individual and structural levels to shape access to healthcare.

### 4.1 Strengths and limitations

The findings from this study should be considered in the context of several strengths and limitations. The total sample size (*n* = 78) was consistent with medium‐sized qualitative survey studies (Braun et al., 2020). The online survey format allowed us to access a geographically dispersed sample of oncology nurses, and elicit a wide range of perspectives, a particular and unique strength of using online surveys as qualitative data collection tools (Braun et al., 2020). Although common in surveys of nurses, with time, workload, and perceived value or relevance of the topic cited as barriers to participation (VanGeest & Johnson, 2011), a low response rate to the email invitations to participate was noted. However, as discussed above, there were overall similarities between participants in this study and those who are members of CANO, which suggests that the sample holds some measure of representativeness. Findings may have been influenced by attrition bias, as less than half (34 of 78) of study participants fully completed the survey; however, the characteristics of partial survey completers and full survey completers were similar. Attrition may have been related to the nature of the questions, with open‐ended questions requiring more time and effort to answer (Dillman et al., 2009), and resulting in higher rates of item nonresponse (Miller & Lambert, 2014).

## 5 CONCLUSION

This examination of nurses' perspectives of access to oncology care among Indigenous Peoples demonstrates how narratives of individualism are embedded within nursing practice and shape how nurses understand and address access to care, while structural barriers to accessing oncology care, including racism, remain relatively invisible. We reiterate that nurses are not intentionally ignoring structural inequities, but rather are taking up broader social discourses that focus their attention at the individual level and work to sustain the invisibility of racism within healthcare settings. However, continually privileging an understanding of equitable healthcare access centered on individual patients risks entrenching these inequities. To begin to rewrite nurses' narratives, nurses must “cast their gaze toward the conditions that perpetuate inequalities” (Cameron et al., 2014, p. E14). Embedding a structural understanding of health and healthcare access into nursing education and practice will open space to move toward redressing inequitable access to oncology care for Indigenous Peoples.

By critically examining nurses' narratives on access to oncology care, we can begin to provide oncology nurses with tools to improve access to care for Indigenous Peoples, and better support them in delivering high‐quality care. Through their interactions with patients and attention to structural determinants of health, nurses have the power to contribute to safe healthcare spaces and address inequities in access to oncology care for Indigenous Peoples in Canada.

## Data Availability

Authors elect to not share data due to privacy and ethical considerations.

## Footnotes

1 In Canada, the term 'Indigenous Peoples' refers to three distinct groups: First Nations, Metis and Inuit. Among First Nations peoples, those who are registered with the federal government under the *Indian Act* are legally referred to as 'registered Indians' or 'status First Nations'.

2 Data for this study was collected in 2019. Since that time, there has been increased attention to racism within North America broadly (e.g., Black Lives Matter protests in the U.S.), and towards Indigenous Peoples in Canadian healthcare settings specifically (e.g., the racist treatment and subsequent death of Joyce Echaquan in a Quebec hospital in 2020). The recent discoveries of unmarked graves on the grounds of residential schools in Canada have also sparked national dialogue regarding the ongoing impacts of racist government policies on the health and wellbeing of Indigenous Peoples. It is possible that awareness of racism in healthcare and oncology settings has grown among nurses as a result.
